# The Choice of Laboratory Methodology Influences Autoantibody Test Results

**DOI:** 10.3389/fimmu.2015.00392

**Published:** 2015-08-03

**Authors:** Johan Rönnelid

**Affiliations:** ^1^Department of Immunology, Genetics and Pathology, Rudbeck Laboratory, Uppsala University, Uppsala, Sweden

**Keywords:** autoantibodies, laboratory diagnosis, immunoassays, rheumatic diseases, quality assessment

## Abstract

During the last 25 years, clinical autoantibody determinations have changed dramatically. Old and slow techniques with high diagnostic specificity have been replaced with automated and faster techniques that most often have a higher diagnostic sensitivity at the expense of a lower diagnostic specificity. Newer techniques are mostly quantitative, allowing for follow-up of autoantibody levels. Whereas the older procedures utilized autoantigens in soluble and native states, most modern techniques rely on autoantigens attached to surfaces, with the risk of exposure of denatured epitopes. Comparisons between antibody measurement techniques can be obtained from the results of external quality assessment programs. As the main objective for external quality assessment is the monitoring of clinical laboratories, they cannot focus on the kind of low-level and often polyreactive sera, which are common in the real world and in which a single definite target response cannot be easily defined. Such common sera are very useful, however, for analysis of differences between autoantibody measurement techniques. The European Consensus Finding Study Group on Autoantibodies has been working with this approach for 28 years.

## Introduction

The current era is truly changing the role of autoimmune diagnostics. The interplay between genes and subsequent environmental exposures leading to autoimmune reactions and ensuing disease has revealed that traditional diagnoses, such as rheumatoid arthritis (RA), can be subdivided into disease entities with divergent genetic and environmental triggers and also be characterized by divergent autoantibody profiles, thus paving the way for larger impact of autoimmunity analyses in diagnostic settings. Indeed, in the previous 1987 American College of Rheumatology (ACR) RA classification criteria, rheumatoid factor (RF) was included as the only autoantibody and could account for 25% (1/4 criteria) of the number of criteria needed to fulfill the classification as RA. In contrast in the more recent joint European League Against Rheumatism (EULAR)/ACR criteria (1), both RF and antibodies against citrullinated proteins/peptides (ACPA) are included and can encompass 50% (3/6 points) of the points needed to fulfill the recent RA classification criteria.

Modern autoantibody detection systems, such as enzyme immunoassays (EIA) including enzyme-linked immunosorbent assay (ELISA), line immunoassay (LIA), and addressable laser bead immunoassay (ALBIA) with the most common brand name Luminex^®^, are also mostly more or less quantitative, whereas older techniques like counter immunoelectrophoresis (CIE) and double immunodiffusion (DID) are qualitative. These possibilities for autoantibody quantification have been utilized in both the new RA (1) and in the tentative new systemic lupus erythematosus (SLE) criteria (2).

Autoantibody analyses also emerge as prognostic markers for therapy selection in already diagnosed patients, where, i.e., RF/ACPA-positive RA patients have a better response to CD20-depletion therapy than do seronegative RA patients.

Given the increasing impact of autoantibody analyses in clinical medicine, it is important to pay attention to the recent and ongoing changes in measurement techniques. There are some distinct differences between the new and old generations of autoantibody tests. The old approaches with precipitation and agglutination techniques detected autoantibody specificities with high diagnostic specificity (3, 4) and used native antigen in soluble form, whereas ELISA, LIA, and ALBIA all use antigen coated to surfaces and thus potentially denatured. This has created problems in, e.g., the diagnosis of diabetes mellitus type I-associated autoantibodies, where for a long time the use of ELISAs was actively discouraged. More recent ELISA kits have, however, improved, however, at least sometimes by presenting more native forms of antigen through the use of capture antibodies or spacers.

The use of recombinant antigens together with solid phase techniques has also enabled and/or increased the detection of non-precipitating autoantibodies such as anti-Ro52 (5).

Another distinction is the diagnostic sensitivity and specificity of the tests. The newer solid phase-based assays almost universally present with higher diagnostic sensitivity as compared to CIE or DID, but at the expense of decreasing diagnostic specificity (6). In a recent study (7), we compared sensitivity and specificity between one each of EIA, LIA, and ALBIA for antibodies against double-stranded DNA (anti-dsDNA) with the traditional *Crithidia luciliae* immune fluorescent test (CLIFT) comparing an SLE cohort (*n* = 178) with a control group consisting of patients with primary Sjögren’s syndrome or RA, and healthy controls (total *n* = 249). CLIFT had the lowest sensitivity and highest specificity, but when the cutoff for the other techniques was increased to the double value of the cut-offs recommended by the manufacturer according to the tentative new SLE criteria (2), they all obtained excellent specificity, but at the expense of a sensitivity somewhat lower than for CLIFT. When 11 SLE patients followed consecutively were evaluated using all four methods, ALBIA associated best with disease activity (7).

A third difference is the use of secondary/detection antibodies. Previously, anti-total immunoglobulin (IgG + IgA + IgM) reagent or polyclonal antibodies raised against total IgG and thus containing anti-light chain antibodies, which cross-reacted with other immunoglobulin isotypes, were often used. More modern test systems almost exclusively use antibodies directed against the Fcγ chain, thus minimizing cross-reactivity against other immunoglobulin isotypes.

Another factor to consider is the fact that modern commercial solid phase assays almost never correct for the background reactivity in individual sera, by, e.g., analyzing sample-specific blank wells in ELISA. We have, e.g., recently reported that patients with active visceral *Leishmania donovani* infection not only react in the commercial anti-CCP2 test but also react equally strong with wells coated with arginine-containing control peptides (8).

Even if percentage specificity differences between different techniques might seem small, such differences will have a large impact on the positive predictive value (PPV) of defining a specific autoantibody in a primary care situation given that SLE and other systemic rheumatic diseases are very uncommon in unscreened primary care populations. True comparisons of PPV between different autoantibody techniques are exceedingly rare, however, and authors often report PPV values based on the relative numbers of patients and controls investigated in their current study and not on the actual frequency of the investigated disease in a relevant real world health care situation.

For a couple of decades, there has been a large number of excellent comparisons performed between different modern assays, i.e., Ref. (6, 9), but new comparative publications do not keep up with the pace of introduction of new tests. Importantly, “true” PPV, a very informative measure, is almost never calculated.

I will discuss some findings of autoantibodies directed against “extractable nuclear antigens” (ENA) and chose this for two reasons. First, anti-nuclear antibody (ANA)-associated anti-ENA reactivities seem, in my view, to be more often afflicted with low-level reactivities that might influence diagnostic performance as compared to other disease-specific autoantibodies, such as anti-tissue transglutaminase in celiac disease, or anti-21-hydroxylase antibodies in Addison’s disease. Second, in contrast to other autoantibodies that are ordered individually, multiple anti-ENA antibodies are usually bundled together in panels with the new EIA, LIA, or ALBIA techniques, and all results are reported in parallel to the clinicians. Thus, any decreases in diagnostic specificity for the individually included anti-ENA autoantibodies will be additive in the clinical situation.

The examples that I will discuss in the next section are derived from Swedish and European external quality assessment programs.

## What Information Can We Get from External Quality Assessment Programs?

During the years 1999–2008, I hosted the Swedish quality assessment program EQUALIS for ANA and anti-ENA in which a total of 23 laboratories from the Scandinavian countries participated. Each laboratory obtained blinded samples four times yearly and reported the analysis results back to EQUALIS. These were thereafter interpreted, and the compiled results were distributed with comments after each distribution. All users were also invited to a yearly national meeting for discussions of the findings. The program still works in essentially the same way.

In 2001, I distributed an IF-ANA negative and anti-SSA/Ro-positive sample to the participating laboratories. Thirteen laboratories performed full testing for ENA, and the five laboratories using DID or LIA reported the expected response whereas all eight laboratories using ELISAs for anti-ENA determination also reported anti-SSB/La. All 13 laboratories reported a negative IF-ANA.

At that time, some laboratories screened for anti-ENA by doing IF-ANA, whereas some laboratories performed screening for anti-ENA ELISA in parallel to the IF-ANA; the latter is currently the common procedure in Sweden. Our results revealed that the IF-ANA screening assay was less sensitive than the follow-up ELISA for anti-SSB/La antibodies, and that this latter reactivity would have passed undetected had the sample followed initial screening using IF-ANA.

Normally, a screening test should be more sensitive than the ensuing follow-up investigations; a typical situation is the screening of blood donors for HIV in which the screening test must detect every single HIV-positive individual at the expense of a number of false positives that will be ruled out in subsequent confirmatory tests. But in the example described above, the situation was the reverse due to changes in technique, as the sensitivities of screening test and follow-up tests over time have moved in opposite directions. The general recommendations for screening dilution for IF-ANA have moved from a combination of 1/40 (corresponding to 32% of healthy controls positive in the original publication) and 1/160 (10) to now recommending a screening dilution corresponding to a specificity of >95% compared to healthy controls, often equivalent to a 1/160 screening dilution (11). The techniques employed for anti-ENA follow-up tests have, conversely in most laboratories, moved from insensitive precipitation techniques to far more sensitive solid phase-based detection systems.

The national Swedish QA program is small and only limited conclusions can be drawn. The United Kingdom National External Quality Assessment Service (UK NEQAS) (12) serves many European immunology laboratories, and their July 2014 distribution for “Antibodies to nuclear and related antigens” had 732 participants with 635 delivering results (an 87% response rate). The distribution covers IF-ANA, anti-dsDNA, anti-centromere responses and anti-ENA treated separately, and I will only focus on the latter. Compiled results from two distributions are shown in Table 1. In the upper part, a sample from 2008 is depicted. There was a considerable consistency among laboratories utilizing modern techniques (ELISA or ALBIA) that reported the determination of anti-RNP in the absolute majority of cases, whereas only one out of very few of the laboratories using precipitation techniques (DID or CIE) could detect anti-RNP. Moreover, a portion of the laboratories using modern procedures but none of the laboratories utilizing precipitation techniques also detected anti-SSA/Ro antibodies. The target response was anti-RNP, implying that the absolute majority of laboratories with modern techniques but only a small fraction of laboratories with the original techniques succeeded in their task. In this context, it is worthwhile to be reminded that the original description mixed connective tissue disease (MCTD) and its association with the autoantibody specificity U1snRNP used neither precipitation techniques nor solid phase immunoassays, but a hemagglutination assay (3).

**Table 1:** **Number of specific anti-ENA responses in two UK NEQAS samples sorted according to the laboratory techniques employed**.

	SSA	SSB	Sm	RNP	Scl70	Jo-1	Negative
**UK NEQAS sample 0821 (target response: anti-RNP)**							
CIE	0	0	0	0	0	0	4
DID	0	0	0	1	0	0	2
ELISA	42	2	5	272	4	1	6
ALBIA	2	0	0	17	0	0	0
**UK NEQAS sample 1142 (target response: anti-SSA/RO + anti-SSB/La)**							
CIE	7	7	0	0	0	0	0
DID	–	–	–	–	–	–	–
ELISA	293	293	1	12	1	2	0
ALBIA	32	32	0	0	0	0	1

*Figures represent number of laboratories delivering a certain response*.

*UK NEQAS, United Kingdom National External Quality Assessment Service; ENA, extractable nuclear antigens; CIE, counter immunoelectrophoresis; DID, double immune diffusion; ELISA, enzyme-linked immunosorbent assay; ALBIA, addressable laser bead immunoassay. No participating laboratories performed DID for the 1142 sample*.

As is known to everyone working with clinical autoimmunity diagnostics, patient sera differ tremendously and whereas some sera exhibit rather high numbers of borderline reactivities, others are strong and monospecific. An example of the latter is shown in the bottom of Table 1. In this serum from the July 2011 UK NEQAS distribution, almost every laboratory, irrespective of technique utilized, detected only the combination of anti-SSA/Ro and anti-SSB/La without the co-occurrence of any other autoantibodies.

External quality assessment services need to determine target responses for all samples, and for that purpose many everyday sera with multiple borderline reactivities are unsuitable. In Table 2, UK NEQAS anti-ENA responses using ELISAs or other EIAs are compiled. Except for the expected simultaneous co-determination of anti-SSB/La in some samples with the target response anti-SSA/Ro and the occasional co-determination of anti-Sm in one sample with the target response anti-RNP, very few laboratories reported positive findings outside the target responses.

**Table 2:** **Number of specific responses for UK NEQAS dispatches for anti-ENA during 1 year (August 2013–July 2014)**.

Sample distribution	SSA/Ro	SSB/La	Sm	RNP	Scl70	Jo-1	Negative
135-1 (August 2013)	296	285	4	3	1	3	0
135-2 (August 2013)	297	40	5	2	0	1	0
136-1 (October 2013)	299	8	2	2	1	1	0
136-2 (October 2013)	5	3	3	3	2	4	94
141-1 (January 2014)	289	9	0	0	0	2	0
141-2 (January 2014)	285	284	0	1	0	3	2
142-1 (March 2014)	12	4	287	277	3	4	3
142-2 (March 2014)	289	172	280	284	2	5	0
143-2 (May 2014)	8	1	0	0	0	0	178
143-2 (May 2014)	296	1	0	0	0	1	1
144-1 (July 2014)	0	0	0	0	269	0	12
144-2 (July 2014)	2	0	17	273	0	0	3

*Only responses for ELISA and other similar EIA are included. Two samples are distributed six times each year. Target responses for the individual distributions are underlined*.

*UK NEQAS, United Kingdom National External Quality Assessment Service; ENA, extractable nuclear antigens; EIA, enzyme immunoassay; ELISA, enzyme-linked immunosorbent assay*.

In order to fulfill their aims of monitoring the performance of clinical laboratories, external QA programs thus need to work with rather straightforward serum samples and rightly do so. But such sera are not the best for evaluation of subtle differences between laboratory techniques.

## The European Consensus Finding Study Group on Autoantibodies

Originally initiated as a workshop in 1988 by Walther J van Venrooij (Nijmegen) and Ravinder N Maini (London), the European Consensus Finding Study Group on Autoantibodies (ECFSG) is now in its 28th year (13). As described in the original publication (14) and still valid, the work aims at investigating the inter-laboratory agreements concerning autoantibody findings in sera containing *unspecified* antibodies, and to gain insight into the variability of autoantibody detection in routine clinical practice.

Each year in December, ECFSG dispatches 10 blinded sera to the (currently 43) participating European laboratories. Before each yearly dispatch, a number of sera have been screened by the laboratories of the steering board members, and the sera finally chosen are deliberately selected to discern differences between laboratories. This means that sera with *disagreement* in the prescreen rounds are often favored. Each participating laboratory delivers their responses electronically in January, results are compiled, and thereafter discussed during a study group session, which takes place every year in conjunction with the annual European Workshop for Rheumatology Research (EWRR), and to which all participating laboratories are invited. The results for each investigated serum are then discussed in a clinical context. It is important to note that no commercial interests are involved as no diagnostic companies or laboratories closely linked to specific diagnostic companies are allowed to participate in the activities. The participating laboratories are advised to deliver detailed data on commercial reagents that have been used, and given the non-commercial setting the relationship between laboratory data and use of specific commercial products can be freely discussed during the study group sessions. The costs for dispatch of sera and prescreen meetings have been covered by EULAR grants.

Reference standards defined previously can sometimes be demonstrated to contain autoantibody specificities undetected at the time of preparation of the standards. For example, in my own laboratory in Uppsala, we have detected anti-SSA/Ro52/TRIM21 autoantibodies in the current CDC reference standard for anti-Jo-1 (15) (unpublished results). Even if such findings do not discredit the use of the reference standard in any way, an early detailed characterization of future reference reagents for additive specificities besides the target specificity is advocated. During recent years, we have therefore included tentative future or newly launched reference reagents among the blinded samples in the ECFSG dispatches, and an anti-dsDNA containing sample evaluated in 2014 will probably be used to manufacture the second WHO anti-dsDNA reference standard.

## Conclusion

The panorama of laboratory techniques used for the determination of autoantibodies has changed dramatically during the last decades. Old laboratory techniques for the determination of autoantibodies (DID, CIE, hemagglutination) are slow and time-consuming and have higher diagnostic specificity but lower diagnostic sensitivity than the modern and faster approaches based on solid phase-coupled antigens (ELISA, LIA, ALBIA). The older techniques were mainly based on soluble antigens in native state, whereas the coupling of the autoantigens to solid phase used in modern technologies confers the theoretical risk of denaturation of the autoantigens. Modern techniques are often more quantitative and better for follow-up purposes. New solid phase-based assays are launched at a high pace. However, the clinical associations described between specific autoantibodies and specific disease entities were performed with the previous generation of autoantibody determination techniques and are not regularly repeated with new test kits. The PPV of new test systems in the clinical situation with rare occurrence of systemic autoimmune diseases is also an issue.

Analysis of results from QA programs can give some insight into how different autoantibody-containing sera react when analyzed using different methods. But given their aims of monitoring laboratories, QA programs cannot focus on serum samples in which results indicate subtle divergences between techniques. The ECFSG is one body deliberately aiming at investigating controversial sera to understand the differences between different laboratory techniques in a clinical context, and more focus on such efforts are needed.

## Conflict of Interest Statement

The author declares that the research was conducted in the absence of any commercial or financial relationships that could be construed as a potential conflict of interest.
